# 25-Hydroxyvitamin D Inhibits Hepatitis C Virus Production in Hepatocellular Carcinoma Cell Line by a Vitamin D Receptor-Independent Mechanism

**DOI:** 10.3390/ijms20092367

**Published:** 2019-05-13

**Authors:** Amiram Ravid, Noa Rapaport, Assaf Issachar, Arie Erman, Larisa Bachmetov, Ran Tur-Kaspa, Romy Zemel

**Affiliations:** 1 Endocrine Immunology Laboratory, Sackler School of Medicine, Tel-Aviv University, Beilinson Hospital, Petah Tikva 49100, Israel; amiramravid@gmail.com; 2 Molecular Hepatology Research Laboratory, Sackler School of Medicine, Tel-Aviv University, Beilinson Hospital, Petah Tikva 49100, Israel; rap.noa@gmail.com (N.R.); larisab@clalit.org.il (L.B.); Ran.Turkaspa@mail.biu.ac.il (R.T.-K.); 3 Liver Institute, Rabin Medical Center, Beilinson Hospital, 49100 Petah Tikva, Israel; assaf@clalit.org.il; 4 Department of Nephrology and Hypertension, Rabin Medical Center, Beilinson Hospital, Petah Tikva 49100, Israel; ranx@bezeqint.net; 5 Faculty of Medicine in the Galilee, Bar-Ilan University, 1311502 Safed, Israel

**Keywords:** vitamin D_3_, 25-hydroxyvitamin D_3_, vitamin D receptor, hepatitis C virus

## Abstract

Previously, we have reported that the active vitamin D metabolite, calcitriol and vitamin D_3_ (cholecalciferol), both remarkably inhibit hepatitis C virus production. The mechanism by which vitamin D_3_ exerts its effect is puzzling due to the low levels of calcitriol produced in vitamin D_3_-treated Huh7.5 cells. In this study, we aimed to explore the mechanism of vitamin D_3_ anti-hepatitis C virus effect. We show that vitamin D_3_ activity is not mediated by its metabolic conversion to calcitriol, but may be due to its primary metabolic product 25(OH)D_3_. This is inferred from the findings that 25(OH)D_3_ could inhibit hepatitis C virus production in our system, and that adequate concentrations needed to exert this effect are produced in Huh7.5 cells treated with vitamin D_3_. Using the CRISPR-Cas9 editing technology to knockout the vitamin D receptor, we found that the antiviral activity of vitamin D_3_ and 25(OH)D_3_ was not impaired in the vitamin D receptor knockout cells. This result indicates that 25(OH)D_3_ anti-hepatitis C virus effect is exerted by a vitamin D receptor-independent mode of action. The possibility that vitamin D_3_ and 25(OH)D_3_, being 3β-hydroxysteroids, affect hepatitis C virus production by direct inhibition of the Hedgehog pathway in a vitamin D receptor-independent manner was ruled out. Taken together, this study proposes a novel mode of action for the anti-hepatitis C virus activity of vitamin D_3_ that is mediated by 25(OH)D_3_ in a vitamin D receptor-independent mechanism.

## 1. Introduction

Vitamin D is increasingly recognized as an important physiological regulator with pleiotropic functions. Cholecalciferol (vitamin D_3_) the 3β-hydroxyl-secosteroid, which is mainly produced in the skin and partly supplied through diet, is an inert compound, which acquires its biological activity through two successive hydroxylations to provide 1α, 25-dihydroxyvitamin D_3_ (calcitriol) [1,2]. The first hydroxylation occurs in the liver, the main source for the major circulating form of vitamin D, 25-hydroxyvitamin D_3_ (25(OH)D_3_). It harbors the physiologically relevant vitamin D 25-hydroxylase (25(OH)ase): CYP2R1, but also three other 25(OH)ases: CYP27A1, CYP2J2, and CYP3A4 [3]. The second hydroxylation occurs in the kidney where 25(OH)D_3_ is metabolized to calcitriol by 25-hydroxyvitamin D 1α-hydroxylase encoded by *Cyp27B1*. Although the kidney was initially thought to be the sole organ expressing CYP27B1, it is now appreciated that its expression in tissues other than the kidney is widespread including in the hepatocarcinoma cell (HCC) line Huh7.5 [4]. Catabolism of calcitriol and 25(OH)D_3_ is governed by 25-hydroxyvitamin-D 24-hydroxylase (24-hydroxylase), encoded by *Cyp24A1*, which is a target to calcitriol action. Calcitriol exerts its biological activities by binding to the vitamin D receptor (VDR), a member of the nuclear receptor family, which forms a heterodimer with RXR and binds to vitamin D responsive elements (VDREs) in the promotor of vitamin D-target genes and mediate their transcription.

However, more than a decade ago the dogma that cholecalciferol is biologically inert was challenged when Biljsma et al. reported that 3β-hydroxysteroid in general, and cholecalciferol in particular, can directly inhibit the Hedgehog (Hh) pathway [5,6,7,8]. Hh signaling is mediated by the membrane protein, patched (Ptch1) which binds and inhibits smoothened (Smo) [9]. Activation of this pathway is initiated by binding of Hh to Ptch1, releasing Smo, and the downstream transcription factor the glioma-associated (Gli) from inhibition. Cholecalciferol was shown to directly bind Smo, thereby preventing the alleviation of Smo and Gli inhibition.

It is also argued that 25(OH)D_3_, which was long regarded as an inactive prohormone, is an agonistic vitamin D receptor ligand at high concentrations. It was shown to have gene regulatory activity with target gene profiles largely matching those of calcitriol [1,2,10].

The vitamin D system is known as essential to the skeletal system; however, during the last 50 years, a multitude of extraskeletal effects have been documented. Recently, a strong association between vitamin D deficiency and the clinical outcome and disease progression of hepatitis C virus (HCV) infections was demonstrated [11]. It was reported that supplementation of vitamin D to pegylated interferon and ribavirin therapy significantly improved sustained virologic response (SVR) rates in patients with chronic HCV infection [12,13,14]. We have recently shown that both calcitriol and vitamin D_3_ remarkably inhibited HCV production in an HCC line [15]. In our study, we found that nanomolar concentrations of calcitriol were required to attain substantial inhibition of HCV production in the Huh7.5 hepatoma cell line, while only picomolar concentrations of calcitriol were produced in these cultures when supplemented with HCV-inhibiting concentrations of vitamin D_3_. In view of this discrepancy, we challenge the presumption that calcitriol is the main and only mediator of the anti-HCV activity of vitamin D_3_ and examine the role of 25(OH)D_3_ as a VDR agonist and of cholecalciferol itself in this activity. Herein, we present evidence that the antiviral activity of vitamin D_3_ is most probably mediated by 25(OH)D_3_ in a VDR-independent mechanism.

## 2. Results

### 2.1. Involvement of Calcitriol in the Anti-HCV Activity of Vitamin D_3_

**Figure 1:**
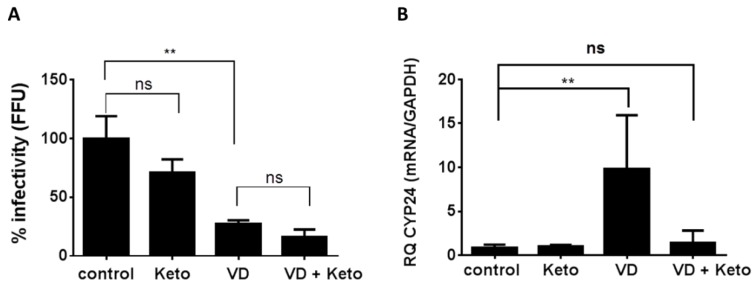
Effect of ketoconazole on the anti-hepatitis C virus (HCV) activity of vitamin D_3_. Huh7.5 cells were treated with vitamin D_3_ (VD) (5 µM), ketoconazole (Keto) (1 µM), or both 3 h prior to infection. Nonsignificant is denoted by ns. (**A**) Inhibition of HCV HJ3-5 virus production, as determined by focus-forming unit (FFU) assay of virus released into cell culture media 24 h post-infection. (**B**) Real-time polymerase chain reaction (PCR) analysis of *Cyp24A1* expression level in Huh7.5 treated cells. A representative of two experiments was performed in triplicates. The results are shown as the relative quantity (RQ) normalized to *Gapdh* mRNA values; the control cells were assigned a value of 1. Statistical significance was calculated by two-tailed Student’s *t* test ** *p* < 0.002, ns—nonsignificant.

**Figure 2:**
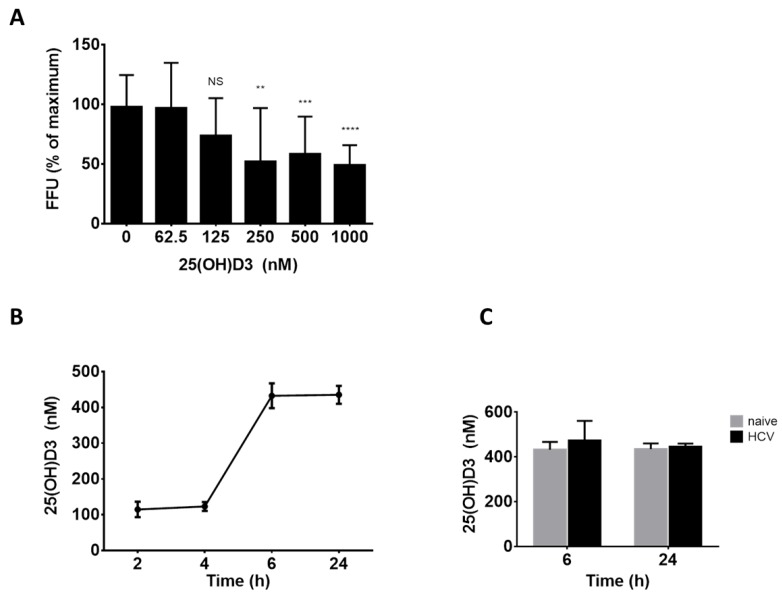
Involvement of 25(OH)D_3_ in mediating vitamin D_3_ anti-HCV effect. (**A**) Inhibition of HCV HJ3-5 virus production as determined by FFU assay of virus released into media following infection and treatment with 25(OH)D_3_ (62.5–1000 nM). Percent of FFU was calculated by comparing with virus released in nontreated cell cultures (0). Mean values ± SD of three different experiments are presented. Statistical significance was calculated by two-tailed Student’s *t* test and is indicated as follows: ** *p* < 0.05, *** *p* < 0.01, **** *p* < 0.0001; ns—nonsignificant. (**B**) ELISA analysis of 25(OH)D_3_ levels produced by noninfected Huh7.5 cells 2–24 h post-treatment with vitamin D (5 μM) and (**C**) HCV infected and noninfected cells 6 and 24 h post-treatment with vitamin D (5 μM). A representative experiment out of two was performed in triplicates.

In our previous study, we found that the inhibition of HCV production by vitamin D_3_ is accompanied by calcitriol generation which results in the induction of the vitamin D target gene *Cyp24A1*. This led us to conclude that the newly produced calcitriol mediates the anti-HCV activity of the vitamin. However, we were troubled by the more than two orders of magnitude difference between calcitriol concentrations required to attain substantial inhibition of HCV production (nanomolar range) and the concentrations of the hormone produced in these cells (picomolar range). It might be argued that the intracellular calcitriol concentrations are higher than those secreted to the medium due to its local production by CYP27B1 expressed in these cells (Figure 2 and Reference [15]). To test this possibility, we aimed to inhibit calcitriol production and examine its intracrine activity. We used the antifungal drug ketoconazole, which is a mixed-function cytochrome P450 inhibitor, that has been shown to inhibit CYP27B1-dependent production of 1,25-dihydroxyvitamin D [16,17]. Cells were pretreated with ketoconazole in the presence or absence of vitamin D_3_ for 3 h and then were infected with the HCV intergenotypic HJ3-5 chimeric virus. As can be seen in Figure 1A, treatment with ketoconazole had no effect on HCV inhibition by vitamin D_3_ as determined by focus-forming unit (FFU) infectious virus assay. To ascertain that ketoconazole inhibited the production of calcitriol, we monitored its effect on the induction of the calcitriol target gene, *Cyp24A1* [8]. Treatment of Huh7.5 cells with ketoconazole abolished *Cyp24A1* mRNA induction (Figure 1B), indicating a markedly decreased production of calcitriol. These results suggest that the anti-HCV effect of vitamin D_3_ is not due to high local concentrations of in situ-produced calcitriol.

### 2.2. The Role of 25(OH)D_3_ as a Direct Mediator of the Antiviral Activity of Vitamin D_3_

Hepatocytes are highly efficient in metabolizing vitamin D_3_ to 25(OH)D_3_ which, at high concentrations (>400 nM), is capable of binding to and activating VDR [12]. Excluding calcitriol in situ production as the mechanism of vitamin D_3_ antiviral activity, we thus examined the possible role of 25(OH)D_3_ generation by the hepatocarcinoma cells in this activity of vitamin D_3_. To this end, cells were treated with increasing concentrations of 25(OH)D_3_ and then infected with HCV. As shown in Figure 2A treatment with 25(OH)D_3_ at concentrations of 250–1000 nM efficiently inhibited HCV production (up to 50%). The inhibition was not due to a cytotoxic effect since treatment with 25(OH)D_3_ did not affect Huh7.5 cell viability (Figure S1A).

We then asked whether the 25(OH)D_3_ concentrations needed to inhibit HCV can be attained in Huh7.5 cell cultures treated with vitamin D_3_. Vitamin D_3_ can potentially be hydroxylated in our cell system by four known human liver vitamin D 25(OH)ases: CYP2R1, CYP27A1, and CYP2J2 and CYP3A4 to produce 25(OH)D_3_ [3]. To evaluate the potential of Huh7.5 cells to produce 25(OH)D_3_, we tested the expression level of the genes encoding for these enzymes. Interestingly, although CYP3A4 is the most abundant CYP450 in human liver [18], it was not detected in the Huh7.5 cells. However, CYP2R1, CYP2J2, and CYP27A1 hydroxylases were highly expressed in Huh7.5 cells (Figure S1B). As expected, and as previously reported, CYP27B1 was expressed at low levels.

These results show that Huh7.5 cells contain the metabolic machinery needed to produce 25(OH)D_3_. To evaluate the concentrations of 25(OH)D_3_ produced in Huh7.5 culture, cells (infected and naïve) were treated with vitamin D (5 µM) and 25(OH)D_3_ in the culture medium, measured by ELISA (Figure 2B). The 6 h post-vitamin D_3_-addition 25(OH)D_3_ concentration reached ~450 nM and leveled off thereafter up to 24 h. The extent of 25(OH)D_3_ production was not influenced by HCV infection (Figure 2C). Since these levels are sufficient to inhibit HCV (Figure 2A), it could be argued that 25(OH)D_3_ may be the active metabolite mediating the anti-HCV activity of vitamin D_3_.

### 2.3. The Role of VDR in the Anti-HCV Activity of Vitamin D_3_

**Figure 3:**
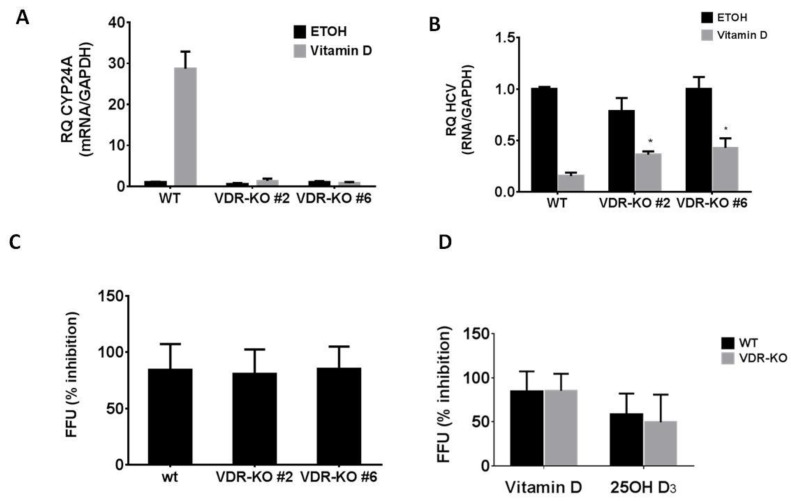
Vitamin D_3_ and 25(OH)D_3_ inhibit HCV production in a VDR-independent manner. (**A**) Real-time PCR analysis of *Cyp24A1* mRNA expression levels in Huh7.5 wild-type (WT) and VDR knockout (VDR-KO) cells after 24 h treatment with vitamin D_3_ (5 µM) compared with nontreated cells. Results are presented as relative quantity (RQ) of the target gene mRNA normalized to *Gapdh* mRNA values; control cells mRNA levels were assigned a value of 1. (**B**) Real-time PCR analysis of HCV RNA expression levels in Huh7.5 WT and VDR-KO cells 24 h post-infection with HCV and treatment with vitamin D_3_ (5 µM) compared with nontreated cells. The results shown are the average of three independent experiments and presented as the relative quantity of the HCV noncoding region normalized to *Gapdh* mRNA values; the control cells were assigned a value of 1. (**C**) Inhibition of HCV HJ3-5 virus production by vitamin D_3_ determined by FFU assay of virus released into media following infection of Huh7.5 and VDR-KO cells. (**D**) Inhibition of HCV HJ3-5 virus production by 25(OH)D_3_ determined by FFU assay of virus released into media following infection of Huh7.5 and VDR-KO #6 cells. The results shown are the average of three independent experiments. Statistical significance was calculated by two-tailed Student’s *t* test (* *p* < 0.0005).

Similar to calcitriol, 25(OH)D_3_ was reported to mediate its effects through VDR engagement. Therefore, we further asked whether the anti-HCV effect of 25(OH)D_3_ and vitamin D_3_ is VDR-dependent. We used CRISPR-CAS9 to produce VDR knockout (VDR-KO) in Huh7.5 cells. To that end, a single guided RNA (sgRNA) targeting the coding sequence of the first translated exon of the *VDR* gene was cloned into the pSpCas9 plasmid, transfected into Huh7.5 cells and analyzed by targeted next-generation sequencing (NGS) (Figure S2). Using limiting dilution, we isolated cell clones with mutated *VDR*. Two clones (clone #6 and clone #2) were subjected to sequence analysis. Clone #2 apparently appeared to contain more than one isolated cell type as indicated by the spectrum of sequences obtained in the analysis (Figure S2Cb) and was considered as a mixed cell population. Sequence analysis of clone #6 revealed a nucleotide insertion which resulted in the putative disruption of the *VDR* open reading frame. VDR–KO was confirmed by the inability of these cells to upregulate *Cyp24A1* expression in response to vitamin D_3_ (Figure 3A). Unexpectedly, vitamin D_3_ and 25(OH)D_3_ were still able to inhibit HCV infection as determined by HCV RNA and by FFU assay (Figure 3B–D), showing that inhibition of HCV production is not mediated by VDR activation.

### 2.4. Involvement of the Hh Pathway in the Anti-HCV Activity of Vitamin D_3_

**Figure 4:**
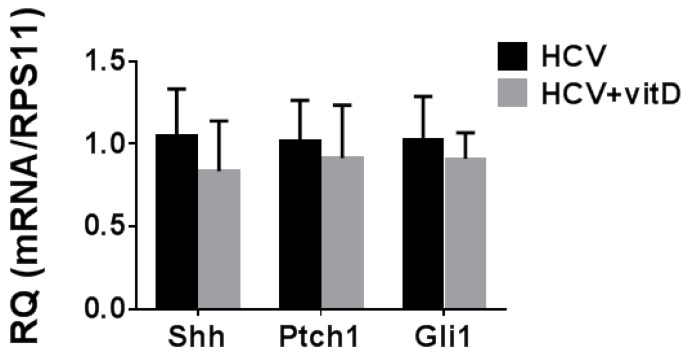
Effect of vitamin D_3_ on mRNA levels of Hedgehog (Hh) pathway target genes in VDR-KO Huh7.5 cells. VDR-KO Huh7.5 cells were treated with vitamin D_3_ (5 µM) three hours prior to infection. Hh target genes expression levels were determined by real-time PCR analysis. Results are presented as relative quantity of target gene mRNA normalized to RPS11 mRNA values. HCV-infected cells were assigned a value of 1. Results are shown as means RQ ± SD of three experiments.

The finding of a VDR-independent mode of action of vitamin D_3_ raised the possibility that the anti-HCV effect is mediated by a direct action of the vitamin. The only direct biological activity reported to date for cholecalciferol is its inhibitory effect on the Hh pathway [9]. An association between HCV replication and Hh pathway activity was reported [19], suggesting that inhibition of the Hh pathway would inhibit HCV replication. The Hh pathway is frequently activated in HCCs and the expression of Hh target genes serves as an indication for pathway activation [20]. Therefore, we measured mRNA levels of the Hh pathway genes and downstream targets *Shh*, *Ptch1*, and *Gli1* in the VDR-KO Huh7.5 cells. We found that the Hh pathway is active in the wild-type (WT) cells (data not shown) as well as in the VDR-KO Huh7.5 cells (Figure 4) in accordance with previous reports [19]. However, as shown in Figure 4, there was no effect of vitamin D_3_ on mRNA expression of these genes in these cells, implying that its antiviral effect in our experimental system is probably not mediated through the Hh pathway.

## 3. Discussion

The physiological activity of vitamin D_3_ is commonly attributed to direct binding of its metabolite 1α,25(OH)_2_D_3_, calcitriol, to the VDR. Calcitriol precursor, 25(OH)D_3_ is regarded as a nonactive prohormone. However, it is now established that 25(OH)D_3_ at supraphysiological concentrations can be a VDR agonist by itself [1]. Not long ago, the dogma that vitamin D_3_ itself is an inert compound was challenged. Vitamin D_3_ as a 3β-hydroxysteroid was shown to inhibit the Hh signaling pathway by directly binding to Smo, one of the elements regulating this pathway [9].

This study aimed to shed light on our puzzling finding that vitamin D_3_, similarly to calcitriol, remarkably inhibited HCV production in HCC cells [15]. We have previously proposed that the antiviral activity of vitamin D_3_ may be mediated by calcitriol produced in Huh7.5 cells by sequential hydroxylation of its 25 and 1 positions. However, examination of the amount of calcitriol generated by the HCV-infected HCC cells revealed that it was too low to account for the antiviral effect of vitamin D_3_. We argued that this antiviral activity may nevertheless be due to the presumed higher local concentrations of the intracellularly produced calcitriol, but could not ignore the possibility that it is mediated through a different mechanism. To distinguish between these possibilities, we inhibited the local conversion of vitamin D_3_ to calcitriol with ketoconazole, a known inhibitor of CYP27B1 [16,17]. We found that ketoconazole did not impair the anti-HCV activity of vitamin D_3_, while inhibiting the intracrine genomic activity of calcitriol as manifested by the lack of induction of its most sensitive target gene, *Cyp24A1*. It should be noted that the use of ketoconazole does not provide a direct evidence for the exclusion of calcitriol as the mediator of vitamin D_3_ antiviral activity. However, the fact that this activity is VDR-independent, strengthens the notion that the antiviral activity of vitamin D_3_ is not mediated by its metabolic conversion to calcitriol.

Another possible mediator of vitamin D_3_ anti-HCV activity is its primary metabolite 25(OH)D_3_. It is well-known that hepatocytes are capable of producing copious amounts of 25(OH)D_3_ that can activate the VDR, making this supposition plausible [1,2]. To explore the validity of this notion, the concentration of 25(OH)D_3_ required to reduce infectious virus production was determined and found to range between 250 nM and 1 μM (Figure 2). These results match perfectly with those of Matsumura et al. [21]. The finding, that such high concentrations of 25(OH)D_3_ can be attained in vitamin D_3_-supplemented Huh7.5 cell cultures, lend further support for this supposition.

Documented direct effects of 25(OH)D_3_ are generally attributed to its VDR-agonistic action. To ascertain that the anti-HCV activity of 25(OH)D_3_ in this study is VDR mediated, its inhibitory effect was examined in VDR-KO cells. We found that knocking out the VDR did not affect the antiviral activity of 25(OH)D_3_ and also of vitamin D_3_ while preventing the induction of *Cyp24A1* by vitamin D_3_. These results indicate that the anti-HCV effects of 25(OH)D_3_ and vitamin D_3_ are exerted by a VDR-independent mode of action.

A VDR-independent mechanism that may account for the anti-HCV effect of vitamin D_3_ is its action on the Hh signaling pathway [9]. This supposition stems from the report showing the involvement of the Hh pathway in HCV replication in vitro [19]. While we found that the Hh pathway is constitutively activated in our cell system as demonstrated by the expression of its downstream target genes, vitamin D_3_ treatment did not affect Hh pathway activity. The resistance of the Hh pathway to vitamin D_3_ treatment may be due to the presence of point mutations in Smo that were shown to prevent the binding of 3β-hydroxysteroids and small molecule inhibitors of the Hh pathway in basal cell carcinoma (BCC) [9]. These results rule out inhibition of the Hh signaling pathway as a mechanism for HCV inhibition by vitamin D_3_.

The current and previous studies provide evidence for the occurrence of several mechanisms of action for the anti-HCV effect of the vitamin D system. We and others have shown that calcitriol inhibited HCV production [15,22,23]. We have found that knocking out the VDR in Huh7.5 cells abolished the anti-HCV activity of calcitriol (data not shown) indicating that this activity is mediated by the VDR. This conclusion is in accord with the report of Yu-Min Lin et al. [23]. It should be noted that two studies failed to show anti-HCV effect of calcitriol at physiological concentrations [21,22]. This discrepancy may stem from variation in VDR expression levels in the specific cell lines used in these studies.

In addition to the antiviral effect of calcitriol, we herein show that 25(OH)D_3_ is capable of inhibiting HCV production in a VDR-independent mechanism. The fact that 25(OH)D_3_ can inhibit HCV was reported previously by Matsumura et al. [21]. Although the authors attribute 25(OH)D_3_ activity to a VDR dependent mode of action, we suppose that, in this system too, 25(OH)D_3_ action may be VDR-independent, since calcitriol was inactive in this system.

We and others [15,22] have shown that treatment with vitamin D_3_ inhibits HCV production. This effect is most probably mediated by its conversion to 25(OH)D_3_. However, it cannot be ruled out that in addition, vitamin D_3_ has an independent direct anti-HCV activity. For example, a liponomic effect may underlie such an anti-HCV activity perturbing the structure of cellular and viral particle membranes which take part in all phases of the HCV life cycle [24]. Knocking out the various 25 vitamin D hydroxylases would provide evidence for a direct effect of cholecalciferol.

Recently, a VDR-independent effect of 25(OH)D_3_ on lipid metabolism was reported [25]. It was shown to impair the activation of the transcription factor sterol regulatory element-binding protein-2 (SREBP2), a master regulator of lipogenesis. This effect was specific to 25(OH)D_3_ and was not shared with vitamin D_3_. SREBP2 is an important transcription factor regulating the synthesis and uptake of lipids including cholesterol [26]. As every step of the virus life cycle is intimately associated with lipid metabolism and cholesterol homeostasis, we are now testing the possibility that vitamin D exerts its effect through regulation of the SREBP pathway.

## 4. Materials and Methods

### 4.1. Reagents

Cholecalciferol, 25-hydroxyvitamin D_3_, and ketoconazole were purchased from Sigma Chemical Co. (St. Louis, MO, USA) and dissolved in absolute ethanol.

### 4.2. Cells

Huh7.5 cells were grown in Dulbecco’s modified Eagle’s medium (Biological Industries, Kibbutz Beit-Haemek, Israel) supplemented with 10% fetal calf serum, penicillin, and streptomycin as described [15].

### 4.3. Inhibition of Infectious Virus Production

Virus assays were carried out with the intergenotypic HJ3-5 chimeric HCV virus. Huh7.5 cells were used for the production of virus stocks and for all assays.

The inhibitory action of vitamin D metabolites on HCV production was assessed essentially as described [15]. Huh7.5 cells were pretreated with vitamin D_3_, 25(OH)D_3_, or the vehicle ethanol for 3 h before infection with the HJ3-5 virus at a multiplicity of infection (moi) of 0.1–0.01. For determination of virus titer, the medium was replaced after 24 h with fresh medium not containing vitamin D_3_ or 25(OH)D_3_, and left for an extra 24 h incubation, in order to eliminate the reagents carry-over. Supernatant fluids were collected from the cell cultures and the titer of infectious virus was determined by the FFU assay, essentially as described [27].

### 4.4. Inhibition of 1,25-Dihydroxyvitamin D Production

Ketoconazole was used for the inhibition of vitamin D_3_ metabolism. Ketoconazole (1 μM) dissolved in ethanol was added concomitantly with vitamin D_3_ to Huh7.5 cells for 3 h before infection with the HJ3-5 virus as above. After 24 h, cells were collected for RNA extraction and analyzed for gene expression.

### 4.5. RNA Isolation and cDNA Synthesis

Total RNA was extracted from cells using EZ-10 DNAaway RNA Miniprep Kit (Bio Basic Inc., Markham, ON, Canada). Total RNA (1 µg) was subjected to reverse transcription (RT) using the qScript cDNA Synthesis Kit (Quantabio, Beverly, MA, USA).

### 4.6. Quantitative Real-time RT-PCR

Real-time RT-PCR assays were performed in the StepOnePlus Real-Time PCR Systems (Applied Biosystems, Foster City, CA, USA), by qScript 1-Step SYBR Green qRT-PCR Kit (Applied Quantabio, Beverly, MA, USA) using gene-specific primer pairs (Table 1).

### 4.7. (OH)D_3_ Determination

The level of 25(OH)D_3_ in cell culture medium was determined by the 25-Hydroxyvitamin DS EIA Assay kit (Immunodiagnostic Systems Ltd., Boldon, UK) according to the manufacturer instructions.

### 4.8. Cell Viability Assay

Cell viability was determined by AlamarBlue Cell Viability Reagent (Invitrogen, Carlsbad, CA, USA) measuring fluorescence intensity in culture supernatants.

### 4.9. VDR Knockout Cells

A guided RNA (gRNA) sequence that targets the genomic sequence in the coding region of the VDR gene in position #63987 (ENSG00000111424) was designed using the in silico prediction tool (http://crispr.mit.edu). The gRNA was designed to be located on an *XhoII* restriction site sequence enabling an easy detection of genome modification (Figure S2). The gRNA sequence: 5’-CGGAACGTGCCCCGGATCTG-3’ was cloned into the pSpCas9(BB)-2A-GFP (PX458) (Addgene, Watertown, MA, USA) plasmid and transfected into the Huh7.5 cells using TransIT Transfection Kit (Mirus Bio LLC, USA). Next-generation sequencing and mutation analysis were performed by Hy Laboratories Ltd. (Israel). In brief, target loci PCR-amplification from the genomic DNA of the cells pool involved using the following primers:

CS1_VDRs-5’-ACACTGACGACATGGTTCTACAAGGGCGAATCATGTATGAGG-3′ and

CS2_VDR as 5’-TACGGTAGCAGAGACTTGGTCTTGCTTCTTCTCCCTCCCTTT-3′

A second PCR was performed on the obtained amplicons using the Access Array index primers for Illumina (Fluidigm) to add the adaptor and index sequences to the sample. The PCR product was purified using AMPure XP beads (Beckman-Coulter), the concentration was measured by Qubit (Invitrogen, USA), and the size determined by Tapestation analysis (Agilent Technologies, USA). The sample was then loaded on the Illumina Miseq and sequenced using a V2-500 cycle kit to generate 250 × 2 paired-end reads. Reads were demultiplexed to generate two FASTQ files and trimmed for quality and adaptor sequences, merged and mapped to the template provided to generate BA/BAI files for the mapping using CLC-Bio software (QIAGEN). More than 90% of the reads were mapped to the template. To measure the frequencies of indels in the target regions, we used the Cas-Analyzer algorithm (http://www.rgenome.net/cas-analyzer/#) [28].

Limiting dilution was performed to select for specific clones with *VDR*-KO gene. Successful transfection was assessed through the detection of green fluorescent protein (GFP)-derived fluorescence in cells. Selection of positive clones was performed by target PCR spanning the gRNA target site in the *VDR* gene (Table 1), digestion with MflI restriction enzyme (Takara) was used for identification of positive clones (Figure S2). Sequence analysis was performed to ascertain VDR-KO clone.

### 4.10. Statistical Analysis

Results are expressed as mean ± SD for replicate cultures. Statistical significance of differences between two experimental groups was determined by the unpaired Student’s *t* test. A value of *p*  < 0.05 was considered statistically significant

## Figures and Tables

**Table 1:** Primer sets used for RT-PCR and PCR.

Accession Number	Primer Name	Sequence (5′→3′)	Product Size (bp)
NM_001128915.1	Cyp24A2-S	ACCCAAAGGAATTGTCCGCA	111
	Cyp24A1-AS	CAAAACGCGATGGGGAGTTC	
NM_024514.4	Cyp2r1-S	TGGAGGCATATCAACTGTGGT	133
	Cyp2r1-AS	GAGTAAGCCTCCCATTTTTGTCA	
NM_000775.4	Cyp2J2-S	TGGACCCCACCAAACTCTCT	153
	Cyp2J2-AS	GGATTGCCTGTGTGCTTT	
NM_000784.4	Cyp27A1-S	GTTCACCACGGAAGGACACC	163
	Cyp27A1-AS	GTTCCCCGAAGCACTCTCTG	
NM_017460.6	Cyp3A4-S	TGTGGGGCTTTTATGATGGT	117
	Cyp3A4-AS	GACCAAAAGGCCTCCGGTTT	
NM_000785.4	Cyp27B1-S	GTGCTAAGACTGTACCCTGTGG	150
	Cyp27B1-AS	ATTTGGCTCTGGGAACTGG	
ENSG00000111424	VDR-S	AGGGCGAATCATGTATGAGG	396
	VDR-AS	TGCTTCTTCTCCCTCCCTTT	
NM_000193.3	SHH-S	GAAACTCCGAGCGATTTAAGGA	228
	SHH-AS	GGCCCTCGTAGTGCAGAGA	
NM_001083603.2	PTCH1-S	TCTTGGTGTTGGTGTGGATG	145
	PTCH1-AS	ATTGCTGATGGACGTGAGG	
NM_005269.2	Gli1-S	CATCAGGGAGGAAAGCAGAC	146
	Gli1-AS	CATTGCCAGTCATTTCCACAC	
NM_001015.5	RPS11-S	GCCCTCAATAGCCTCCTTGG	149
	RPS11-AS	TTCAGACTGAGCGTGCCTAC	
NM_002046.7	GAPDH-S	GAAGGTGAAGGTCGGAGTC	226
	GAPDH-AS	GAAGATGGTGATGGGATTTC	

## Supplementary Materials

Supplementary materials can be found at https://www.mdpi.com/1422-0067/20/9/2367/s1.
