# Supplementary material for: 25-Hydroxyvitamin D Inhibits Hepatitis C Virus Production in Hepatocellular Carcinoma Cell Line by a Vitamin D Receptor-Independent Mechanism
Source: Int J Mol Sci. 2019 May 13;20(9):2367. doi: 10.3390/ijms20092367 (PMC6539257; doi:10.3390/ijms20092367)
Supplement: Supplementary file 1 [file ijms-20-02367-s001.pdf]

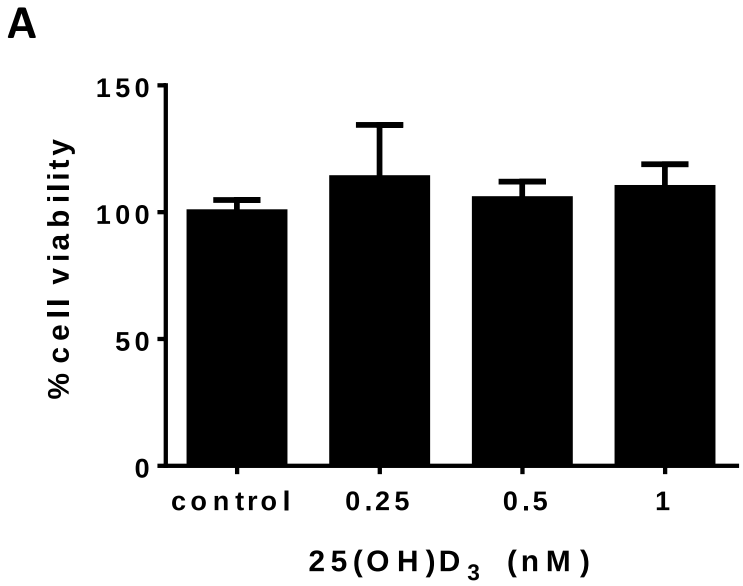

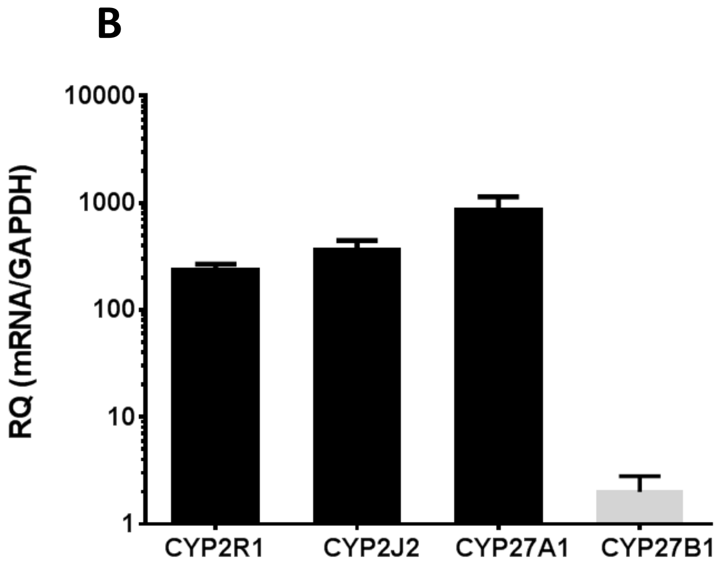

Figure S1. Effect of 25(OH)D_3 on Huh7.5 cell viability.

Huh7.5 cells treated with different concentration of 25(OH)D3 (12.5-1000nM) for 24h. Cell viability was determined by AalamarBlue Cell Viability Reagent measuring fluorescence intensity in culture supernatants. Percent of inhibition and percent of viable cells was determined compared to non-treated cells set as 100%. (B) Real-time PCR analysis of vitamin D 25-hydroxylases (25(OH)ases RNA expression levels in Huh7.5 cells. Results are presented as relative quantity of the target gene normalized to GAPDH mRNA values. CYP27B1 was assigned a value of 1 and results are normalized to it. Results are shown as means relative quantity ± SD.

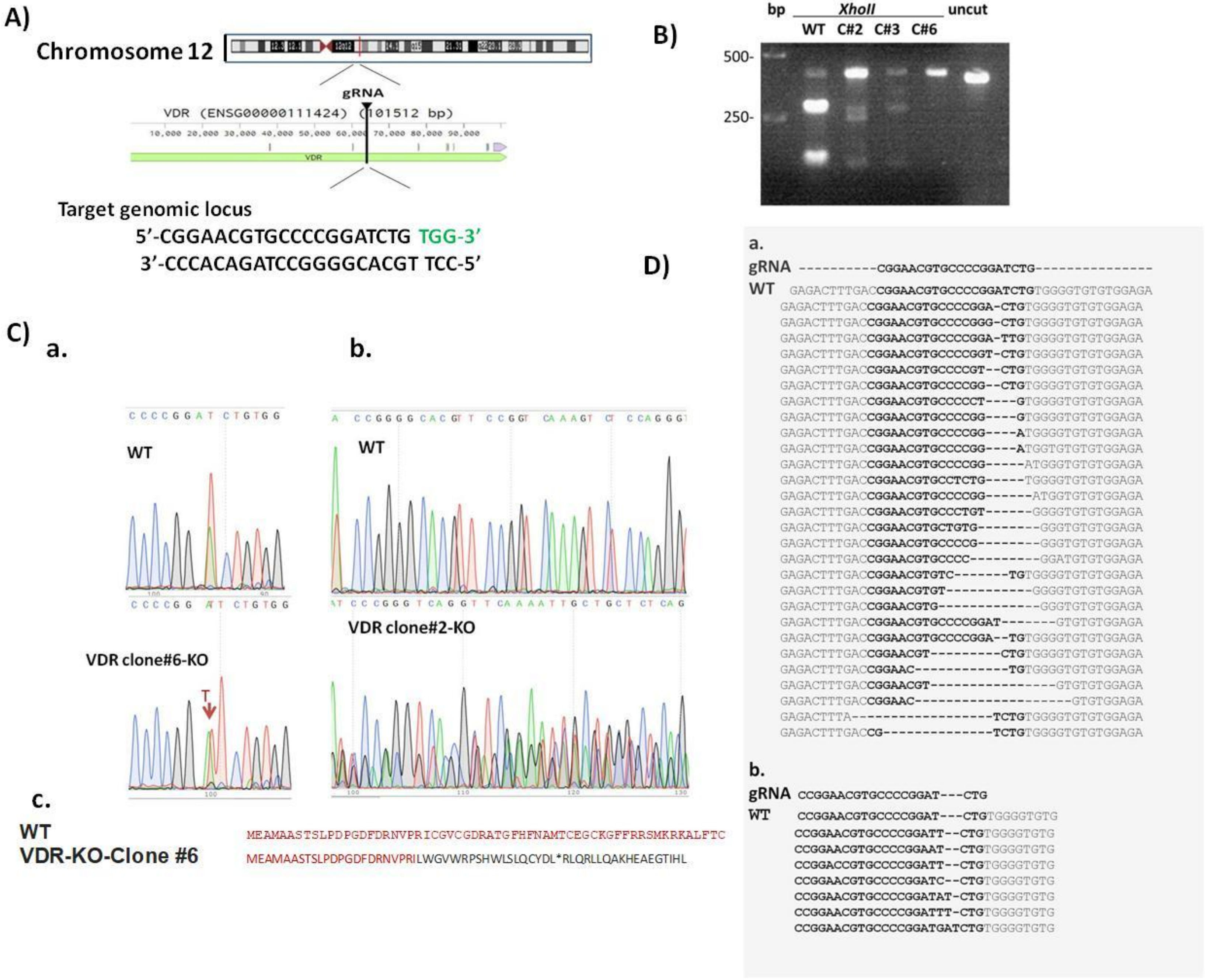
A)
Chromosome 12
gRNA
VDR (ENSG00000111424) (101512 bp)
10,000 20,000 30,000 40,000 50,000 60,000 70,000 80,000 90,000
VDR
Target genomic locus
5'-CGGAACGTGCCCCGGATCTG TGG-3'
3'-CCCACAGATCCGGGGCACGT TCC-5'
B)
bp
XhoII
uncut
WT C#2 C#3 C#6
500-
250-
C) a.
b.
WT
VDR clone#6-KO
T
WT
VDR clone#2-KO
c.
WT
VDR-KO-Clone #6
MEAMAASTSLPDPGDFDRNVPRICGVCGDRATGFHFNAMTCEGCKGFFRRSMKRKALFTC
MEAMAASTSLPDPGDFDRNVPRILWGVWRPSHWLSLQCYDL*RLQRLLQAKHEAEGTIHL
D)
a.
gRNA ----------CGGAACGTGCCCCGGATCTG---------------
WT GAGACTTTGACCGGAACGTGCCCCGGATCTGTGGGGTGTGTGGAGA
GAGACTTTGACCGGAACGTGCCCCGGA-CTGTGGGGTGTGTGGAGA
GAGACTTTGACCGGAACGTGCCCCGGG-CTGTGGGGTGTGTGGAGA
GAGACTTTGACCGGAACGTGCCCCGGA-TTGTGGGGTGTGTGGAGA
GAGACTTTGACCGGAACGTGCCCCGGT-CTGTGGGGTGTGTGGAGA
GAGACTTTGACCGGAACGTGCCCCGT--CTGTGGGGTGTGTGGAGA
GAGACTTTGACCGGAACGTGCCCCGG--CTGTGGGGTGTGTGGAGA
GAGACTTTGACCGGAACGTGCCCCCT----GTGGGGTGTGTGGAGA
GAGACTTTGACCGGAACGTGCCCCGG----GTGGGGTGTGTGGAGA
GAGACTTTGACCGGAACGTGCCCCGG----ATGGGGTGTGTGGAGA
GAGACTTTGACCGGAACGTGCCCCGG----ATGGTGTGTGTGGAGA
GAGACTTTGACCGGAACGTGCCCCGG-----ATGGGTGTGTGGAGA
GAGACTTTGACCGGAACGTGCCTCTG-----TGGGGTGTGTGGAGA
GAGACTTTGACCGGAACGTGCCCCGG------ATGGTGTGTGGAGA
GAGACTTTGACCGGAACGTGCCCTGT------GGGGTGTGTGGAGA
GAGACTTTGACCGGAACGTGCTGTG--------GGGTGTGTGGAGA
GAGACTTTGACCGGAACGTGCCCCG--------GGGTGTGTGGAGA
GAGACTTTGACCGGAACGTGCCCC---------GGATGTGTGGAGA
GAGACTTTGACCGGAACGTGTC-------TGTGGGGTGTGTGGAGA
GAGACTTTGACCGGAACGTGT-----------GGGGTGTGTGGAGA
GAGACTTTGACCGGAACGTG------------GGGTGTGTGGAGA
GAGACTTTGACCGGAACGTGCCCCGGAT-------GTGTGTGGAGA
GAGACTTTGACCGGAACGTGCCCCGGA--TGTGGGGTGTGTGGAGA
GAGACTTTGACCGGAACGT---------CTGTGGGGTGTGTGGAGA
GAGACTTTGACCGGAAC------------TGTGGGGTGTGTGGAGA
GAGACTTTGACCGGAACGT----------------GTGTGTGGAGA
GAGACTTTGACCGGAAC------------------GTGTGGAGA
GAGACTTTA-----------------TCTGTGGGGTGTGTGGAGA
GAGACTTTGACCG-------------TCTGTGGGGTGTGTGGAGA
b.
gRNA CCGGAACGTGCCCCGGAT---CTG
WT CCGGAACGTGCCCCGGAT---CTGTGGGGTGTG
CCGGAACGTGCCCCGGATT--CTGTGGGGTGTG
CCGGAACGTGCCCCGGAAT--CTGTGGGGTGTG
CCGGACCGTGCCCCGGATT--CTGTGGGGTGTG
CCGGAACGTGCCCCGGATC--CTGTGGGGTGTG
CCGGAACGTGCCCCGGATAT-CTGTGGGGTGTG
CCGGAACGTGCCCCGGATTT-CTGTGGGGTGTG
CCGGAACGTGCCCCGGATGATCTGTGGGGTGTG

Figure S2. Outline of the generation of VDR-knockout (KO) Huh7.5.cells

(A) Schematic presentation of the target genomic locus of the human VDR gene. A boxed sequence indicates the site targeted by the guide RNA designed in this study. PAM; protospacer adjacent motif depicted in green (B) Gel electrophoresis of VDR target specific PCR of WT and selected clones (2,3 and 6) after digestion with MflI restriction enzyme. (C) DNA sequencing histogram of a. VDR #6 mutant and WT gene, the red arrows indicated the insertion positions; b. VDR #2 mutant and WT gene. c. Putative protein translation of the WT and the mutated seq. (VDR #6). (D) Sequence alignment of target regions of VDR gene sequences in mutated cell pool based on deep sequencing and analysis with the Cas-Analyzer analysis, gRNA position is indicated, multiple sequence alignment was performed using MULTALIN program (https://npsa-prabi.ibcp.fr/cgi-bin/npsa_automat.pl?page=/NPSA/npsa_multalinan.html) a) deletions b) insertions
